# Sea Lice (*Lepeophtheirus salmonis*) Infestation Reduces the Ability of Peripheral Blood Monocytic Cells (PBMCs) to Respond to and Control Replication of Salmonid Alphavirus in Atlantic Salmon (*Salmo salar* L.)

**DOI:** 10.3390/v12121450

**Published:** 2020-12-16

**Authors:** Amr A. A. Gamil, Koestan Gadan, Elisabeth Gislefoss, Øystein Evensen

**Affiliations:** Department of Paraclinical Sciences, Faculty of Veterinary Medicine, Norwegian University of Life Sciences, P.O. Box 369, 0102 Oslo, Norway; amr.gamil@nmbu.no (A.A.A.G.); koestan.gadan@nmbu.no (K.G.); elisabeth.gislefoss@nmbu.no (E.G.)

**Keywords:** *Lepeophtheirus salmonis*, innate immune responses, salmonid alphavirus, interaction

## Abstract

Here we have studied the impact of lice (*Lepeophtheirus salmonis*) infestation of donor fish on the ability of isolated peripheral blood monocytes (PBMCs) to control the replication of salmonid alphavirus (SAV) ex vivo. PBMCs were collected by Percoll gradients at eight and nine weeks post copepodid infestation of Atlantic salmon post smolt. Uninfested fish were controls. PBMCs were then infected ex vivo with SAV (subtype 3), and samples were collected for analysis at two, four, and six days post virus infection. Virus titer in the supernatant was assayed in CHH-1 cells, and in addition, the relative expression of the virus structural protein E2 and selected host antiviral genes, IRF9, ISG15, Mx, and IFIT5, were assayed using real-time PCR. Significantly higher virus replication was detected in cells collected from lice-infested fish compared to controls. Higher virus titer coincided with an inability to upregulate the expression of different immune genes, IFIT5, IRF9, and Mx. These findings point towards compromised ability of PBMCs from lice-infested fish to control virus replication, and, to our knowledge, is the first report showing the direct effect of lice infestation on the interplay between viruses and immune cells. There is a possible impact on the dynamic spread of viral diseases in the aquatic environment.

## 1. Introduction

Sea lice (*Lepeophtheirus salmonis*) infestations remain one of the major challenges for the salmon industry, and the annual costs of treatment exceeded 5 billion NOK in 2018 [1,2,3]. The life cycle of the sea lice starts with three free swimming stages, nauplius I, nauplius II, and copepodids, where the latter is the infective stage. The copepodids attach to the host and go through two chalimus and two pre-adult stages before reaching the adult stage [4]. During the chalimus stages, the parasite attaches itself to the fish using a structure called the frontal filament, and has limited mobility, whereas the pre-adult and adult stages move freely. While on the hosts, the parasite feeds on mucous and skin tissue, causing wounds and, in severe cases, death.

Immune modulation where innate (inflammatory) host responses are downplayed is believed to be one of the main factors that enable the sea lice to stay on the hosts for a prolonged period [5,6,7,8,9,10]. This immune modulation, in addition to the wounds and stress induced by the lice infestation, may facilitate secondary infections and increase the host’s susceptibility to other pathogens. Until recently, studies on sea lice interaction with the host has been focused mainly on understanding early local and systemic inflammatory responses [5,6,7,8,11], while the interplay between lice infestation and infection with other pathogens has only recently been brought to the fore. The studies conducted thus far indicate that lice infestation plays a role in modulating infection with both viral and bacterial pathogens [3,12], and may also impact vaccination efficacy [13].

Infection with SPDV/salmonid alphavirus (SAV) causes pancreas disease in Atlantic salmon. The disease is classified as a notifiable disease in Norway, and is one of the important causes of losses in salmon farming in Norway and the UK [14]. SAV is a single stranded, positive polarity RNA virus belonging to the genus Alphavirus in the family *Togaviridae* [15,16]. SAV are grouped into six subtypes (SAV-1 to SAV-6) [17], and both SAV-2 and SAV-3 are detected in Norway, with SAV-3 causing higher mortality in experimental challenges [18,19]. SAVs have been found in lice in earlier studies [20], but the importance of these findings is not known. Here we have explored the interplay between lice and SAV-3 infection, where salmon were experimentally infested with *Lepeophtheirus salmonis* copepodids for 8–9 weeks until the lice developed into the pre-adult lice stage. Thereafter, fish were used as donors of peripheral mononuclear cells that were subsequently infected in vitro with SAV-3. Non-infested fish served as donors of PBMCs, and cells were also infected with SAV-3.

## 2. Materials and Methods

### 2.1. Ethical Statement

The experiment was performed in an approved experimental facility by the Norwegian Animal Research Authority. All of the fish handling and the experimental procedures were performed by approved personnel and in accordance with the laws and regulations governing the experimental use of live animals in Norway.

### 2.2. Cell Lines

Chum salmon heart cells CHH-1 (ECACC 92110412) were maintained in L-15 media with Glutamax^®^ (Gibco, Sigma Aldrich, St. Louis, MO, USA) supplemented with 10% FBS (Sigma Aldrich, St. Louis, MO, USA [21]). For maintenance, cells were grown at 20 °C.

### 2.3. Virus Propagation

Isolate SAV-3-H10 was used in the experiments [22]. For propagation, the virus was inoculated into 70–80% confluent CHH-1 cells and incubated at 15 °C until full CPE. The supernatant containing the virus was then harvested, centrifuged at 2500 rpm (950× *g*) at 4 °C for 10 min to remove debris, and then transferred to new tubes and kept at −80 °C until used. The concentration of the virus was estimated by titration in 96 well plates (Falcon, Sigma Aldrich, St. Louis, MO, USA) containing 90–100% confluent CHH-1 cells using the TCID_50_ method [23].

### 2.4. Experimental Design

#### 2.4.1. In Vivo Experiment

Atlantic salmon (*Salmo salar* L.) post smolts (300 g, *n* = 20), reared at the research facility of the Norwegian Institute for Water Research (Solbergstrand), were infested with sea lice copepodids at an infestation density of 70 copepodids/fish. The challenge was carried out by stopping the water flow. Copepodids were added to the stagnant water that was oxygenated throughout the challenge. Oxygen was kept above 8 mg/L throughout the challenge. After 30 min, water flow was resumed, and oxygenation was stopped. A separate group of donors representing non-infested fish was included.

Fish were kept in 500 L tanks, and water quality and oxygen levels were monitored daily during the challenge period. Three fish were sampled at eight weeks and an additional three fish at nine weeks post copepodid challenge in the infested group (*n* = 6 total), and the same number of fish were collected at the same time points for non-infested groups (*n* = 6 total). The fish were anesthetized, and blood samples were collected in heparinized vacutainer by severing *Vena caudalis*. Blood samples were kept on ice, then transported to the laboratory of the Faculty of Veterinary Medicine, Norwegian University of Life Sciences, and processed further (see below).

#### 2.4.2. Ex Vivo Experiments

Blood samples collected from lice infested (*n* = 6) and non-infested fish (*n* = 6), as described above, were diluted with an equal volume of phosphate buffered saline (PBS) containing 2% FBS. Cells were then isolated using 37%/54% Percoll (Sigma Aldrich, St. Louis, MO, USA) gradient, as previously described [24]. After isolation, cells were counted and seeded in a 12-well plate (Corning, Amsterdam, The Netherlands) at a density of two million cells/well. The media used was L-15 media with Glutamax^®^ (Gibco, Waltham, MA, USA), supplemented with 1% fetal bovine serum (FBS, Sigma Aldrich), 2% heat inactivated salmon serum, and a penicillin (100 U/mL) streptomycin (100 μg/mL) mixture (Sigma Aldrich, St. Louis, MO, USA). One day after seeding, cells were either infected with 10^7^ TCID_50_ of SAV-3-H10 isolate or left untreated. Three wells were seeded per fish, two were infected with the virus, and one was left as the uninfected control. Cells were sampled two, four, and six days post infection, and cell cultures were examined together with uninfected control cells. At the time of sampling, supernatants were collected from infected cells and titrated in CHH-1 cells [22]. Parallels of infected and uninfected cells from each fish were lysed by adding RLT buffer to the wells, and RNA was extracted using an RNeasy kit (Qiagen, Hilden, Germany). The two infected wells from each fish were pooled to get enough RNA.

Following RNA extraction, cDNA synthesis was performed using the Transcriptor First Strand CDNA synthesis kit (Roche, Basel, Switzerland). Thereafter, quantitative PCR was performed in 96-well plates using the LightCycler 480 system (Roche). For each reaction, 3 μL of cDNA (diluted 1:3) was mixed with 5 pmol of gene-specific primers and 5 μL of LightCycler 480 SYBR green I master mix (Roche). The final concentration was adjusted to 10 μL using RNase free water. Previously published primers for the virus SAV-3-E2 and the host β-actin, ISG15, Mx, and IFIT5 genes [25], in addition to the following IRF9 gene primers (IRF9-F: AAGGAGGAGGAGGTTGTGGT; IRF9-R: CGAACTGGTCTTGTTGGATG), were used. The following cycling conditions were used: denaturation 94 °C for 10 s; annealing 60 °C for 10 s; elongation 72 °C for 15 s. The results were analyzed following the ∆∆CT relative quantification approach [26] using β-actin as reference gene. The efficiencies of the primers used were determined as follows; for β-actin 103%, IRF-9 95.9%, Mx 98.2%, SAV 98.6%, ISG15 96.7%, and IFIT5 91.9%.

### 2.5. Graphics and Statistical Analysis

Statistical analysis and graphs were made using Stata15. Normality (for residuals) was tested using the Shapiro–Wilk test, and differences between groups (PBMCs donors being lice-infested or not) were analyzed using a Kruskal–Wallis equality-of-populations rank test to check differences in virus replication levels/mRNA expression and responses to SAV-3 infection ex vivo over the course of infection (2–6 days post infection). One outlier was removed for ISG15 and IFIT5 at day six.

## 3. Results

**Figure 1:**
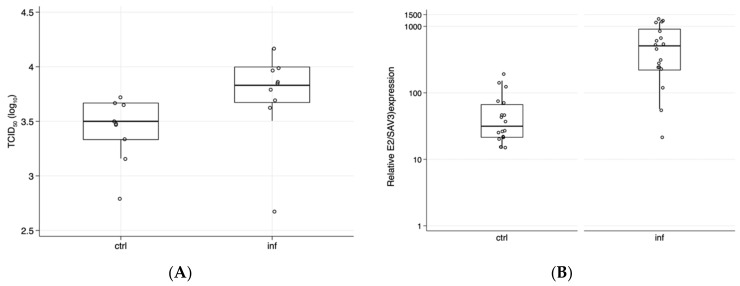
Replication of SAV-3 in PBMCs from non-infested and lice-infested fish. PBMCs were isolated using Percoll gradient and infected with SAV-3. Panel (**A**): Virus titers in supernatants compiled for all time points post infection. Bars represent log TCID_50_/_mL_ ± 25/75% percentiles, and titers for individual cell cultures from individual fish are shown, with a significant difference between groups, *p* = 0.016 (*n* = three fish per group, sampled at three time points and combined). Panel (**B**): Relative mRNA expression of SAV-3-E2 in cells obtained from lice non-infested (ctrl) and infested (inf) fish, three fish with two parallel cell cultures and three time points combined for each of the ctrl and inf groups (*n* = 18 per group, *p* = 0.0001). Results are shown as a normalized expression to virus uninfected cells from each fish using the ddCt method ± 25/75% percentiles and results for individual fish. Whiskers show the 90% percentile.

The total lice count in the infested fish averaged 18.7 and 36 lice/fish by the time blood was sampled at eight and nine weeks post challenge, respectively, indicating a successful lice infection, all of which were at the pre-adult/adult lice stages. Fish were collected from a group of fish (*n* = 12) with an average lice number of 30.1 (±7.1 SD). PBMCs obtained from lice infested and non-infested fish were infected with SAV-3, and virus replication was monitored in supernatants and in cells. Across time points, the virus titers (*p* = 0.016, Kruskal–Wallis test) were significantly higher in supernatants taken from lice-infested fish compared to those obtained from non-infested (control) fish (Figure 1A). When cells from lice-infested and non-infested fish were compared at two, four, and six dpi, there was no statistical difference at two and four dpi (*p* = 0.513 and *p* = 0.121), while a significant difference was detected at six dpi (*p* = 0.046).

There was a significant difference in E2 expression (mRNA) between the two groups across time points (Figure 1B, *p* = 0.0001). The difference in expression was observed for each time point post infection, two dpi (*p* = 0.004), and at four and six dpi (*p* = 0.020 and *p* = 0.0039, respectively). These data align with previous reports showing that lice infection renders fish more susceptible to viral and bacterial infections [3,12]. Studies have shown that the proportion of fish positive for infectious salmon anemia virus by cell culture and PCR analysis was higher in sea lice infested groups [3]. While the number of lice/fish in our study was high compared to what is found under natural conditions when strict lice control is imposed (<0.5 mature female lice on average in the population), it is comparable to the lice challenge pressure in the referred study [3] based on an experimental challenge with *Lepeophtheirus salmonis* and with peak mobile lice numbers of 55. While the number of lice under a normal production environment would not reach >50 lice/fish, 10–20 movable lice can be found in individual fish. The timing of PBMCs collection relative to lice infection is also comparable to the study by Barker et al. [3], where they tested ISAV/lice interaction at 37 and 51 days post infection. Jointly, these findings indicate that lice infestation results in a reduced ability to control virus replication.

**Figure 2:**
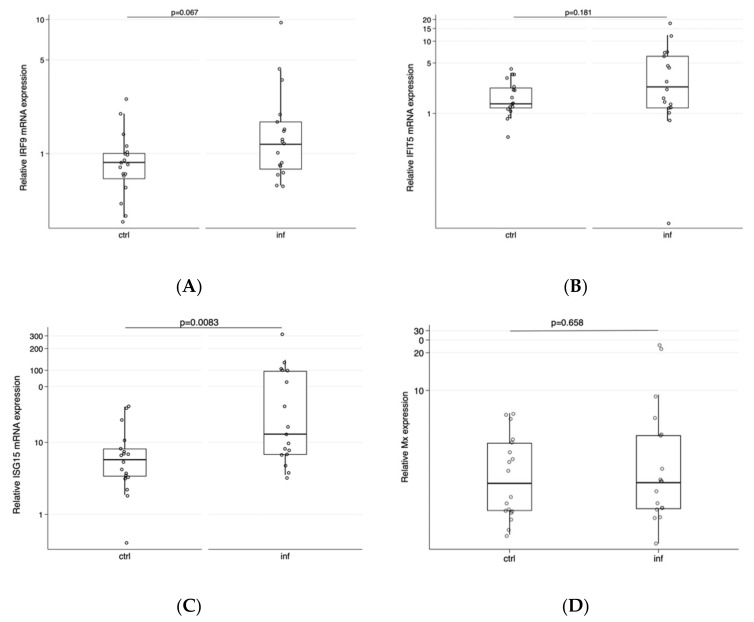
Relative mRNA expression of (**A**) IRF9, (**B**) IFIT5, (**C**) ISG15, and (**D**) Mx in cells obtained from non-infested (ctrl, *n* = 3) and lice-infested (inf, *n* = 3) fish followed over a period of six days in vitro after SAV-3 infection. Bars represent the normalized expression in cells obtained from different individuals at different time points relative to cells not infected with the virus using the ddCt approach and median (25/75 percentile) and 90% percentile for whiskers; *p*-values for differences between controls (ctrl) and infected (inf) are given above the graphs.

To better understand the potential underlying responses that could explain the differences observed, we focused on potential compromised antiviral responses, focusing on the up- and downstream of interferon-induced responses. For all investigated genes except for ISG-15, we found no difference in gene expression in cells obtained from lice-infested fish compared PBMCs from non-infested fish (Figure 2).

Lice immunomodulate their hosts by secreting a complex cocktail of bioactive compounds [5]. The released secretory/excretory products by the lice is found to be high in Atlantic salmon, but can vary between different host species [27]. Concordant with this observation, Atlantic salmon is found to be the most susceptible salmonid species [28,29], and lice infections downplay innate responses locally in skin [6] and systemically downregulate innate responses [8]. Macrophage/monocytes are important effector cells in the immune system, and their interaction with viruses may be crucial for the success of virus infection. They contribute to the inhibition of virus replication through cytokine production (autocrine/paracrine effect) and orchestration of the inflammatory and adaptive immune responses [30]. In some cases, however, they can promote the infection by supporting initial virus replication (primary replication sites) and facilitating the spread to the target organ(s) [31]. Here we found that PBMCs isolated from salmon with heavy lice infestation result in an inability to upregulate antiviral genes at the early time of SAV-3 infection in vitro, resulting in higher level of virus replication shown as virus titer and E2 mRNA expression. The underlying mechanisms that impact the ability to limit virus replication are not understood, but, to our knowledge, this is the first time that the interplay of lice infestation and virus infections in salmon has been addressed. The findings highlight a possible impact of lice infestation on SAV-3 infections and, possibly, virus infections in general in farmed salmon.

While the underlying cellular mechanisms are not understood, it has been shown that lice secretions can modulate immune responses in cell cultures and primary head kidney macrophages [32], and thus a direct (or indirect) effect of lice secretions cannot be excluded. From studies conducted in higher vertebrates, M1 polarization of macrophages favors the establishment of virus infections [33]. Preliminary data obtained in our laboratory have shown a tendency towards M1 polarization in salmon post lice infestation (unpublished data). Hence, immunomodulatory effects may have played a role here, but more detailed studies are required to address this issue.

Lice infestation induces stress and leads to an increase in cortisol and heat shock proteins (HSPs) 60, 70, and 90 [34,35]. HSPs are involved in different signaling cascades, and can play roles in modulating inflammatory responses [36]. Infection with sea lice has been shown to result in increased plasma cortisol concentrations, also related to the lice number on the fish [37], but lice infestation by itself (in the absence of stress) has been shown to downplay important innate immune responses [38]. The effect of cortisol on inflammatory responses and virus infection is also well-understood in salmon, and chronically elevated cortisol levels modulate the immune responses and prevalence of infection with infectious pancreatic necrosis virus [39]. Responses to LPS stimulation in isolated macrophages from experimentally stressed fish with elevated plasma cortisol levels are poorer compared to non-stressed controls [40]. While we did not include an assessment of plasma cortisol in this study, it cannot be ruled out that the high lice infestation levels (total of 52/fish) will result in high cortisol levels and thus have an impact on virus replication levels.
